# Antagonistic Regulation of Flowering Time through Distinct Regulatory Subunits of Protein Phosphatase 2A

**DOI:** 10.1371/journal.pone.0067987

**Published:** 2013-07-26

**Authors:** Behzad Heidari, Dugassa Nemie-Feyissa, Saijaliisa Kangasjärvi, Cathrine Lillo

**Affiliations:** 1 University of Stavanger, Centre for Organelle Research, Faculty of Science and Technology, Stavanger, Norway; 2 Molecular Plant Biology, Department of Biochemistry and Food Chemistry, University of Turku, Turku, Finland; University of Nottingham, United Kingdom

## Abstract

Protein phosphatase 2A (PP2A) consists of three types of subunits: a catalytic (C), a scaffolding (A), and a regulatory (B) subunit. In *Arabidopsis thaliana* and other organisms the regulatory B subunits are divided into at least three non-related groups, B55, B’ and B″. Flowering time in plants mutated in *B55* or *B'* genes were investigated in this work. The *PP2A-b55α* and *PP2A-b55β* (knockout) lines showed earlier flowering than WT, whereas a *PP2A-b’γ* (knockdown) line showed late flowering. Average advancements of flowering in *PP2A-b55* mutants were 3.4 days in continuous light, 6.6 days in 12 h days, and 8.2 days in 8 h days. Average delays in the *PP2A-b’γ* mutant line were 7.1 days in 16 h days and 4.7 days in 8 h days. Expression of marker genes of genetically distinct flowering pathways (*CO, FLC, MYB33, SPL3*), and the floral integrator (*FT, SOC1*) were tested in WT, pp2a mutants, and two known flowering time mutants *elf6* and *edm2*. The results are compatible with B55 acting at and/or downstream of the floral integrator, in a non-identified pathway. *B’ γ* was involved in repression of *FLC*, the main flowering repressor gene. For *B’γ* the results are consistent with the subunit being a component in the major autonomous flowering pathway. In conclusion PP2A is both a positive and negative regulator of flowering time, depending on the type of regulatory subunit involved.

## Introduction

Protein phosphatase 2A (PP2A) is conserved among eukaryotes, and is vital for growth and development, but very little is known concerning functions of specific subunits of PP2A in plants [1–5]. PP2A complexes are composed of three different types of proteins; a catalytic (C), a scaffolding (A), and a regulatory (B) subunit. In *Arabidopsis* , at least seventeen regulatory B subunits are present, and these subunits are believed to be responsible for substrate specificity and cellular localization of the PP2A complexes, hence largely account for the diverse functions of PP2As [1,6–10]. In plants, the B subunits are divided into three main, non-related subgroups: B55 (also called B), B', and B″. In *Arabidopsis* , the B55 subgroup consists of only two members, Bα and Bβ. Our recent work related to nitrogen metabolism had brought to our attention that although the single mutant lines looked normal, these two genes are essential for survival because the *pp2a-bα* x *pp2a-bβ* double knockout was embryo lethal [11]. We decided to further study these genes in relation to growth and development, especially flowering time. Experiments with the *pp2a-bα* and *pp2a-bβ* mutants soon revealed that these mutants were early flowering. We had also noticed in introductory flowering time experiments that some *pp2a* mutants were late flowering. Mutants of the *B*’ η subfamily i.e. *b*’ γ, *b*’ η [6,12] and *b*’ θ (unpublished data) were late flowering. Since *b*’ γ had the most striking phenotype in respect to flowering time, this mutant was chosen to be included in the more detailed studies to reveal underlying molecular mechanisms. B'γ provides a link between developmental regulation and and stress signaling because B’ γ also plays a key role in controlling the extent of defence reactions against different types of plant pathogens [10,12].

**Figure 1:**
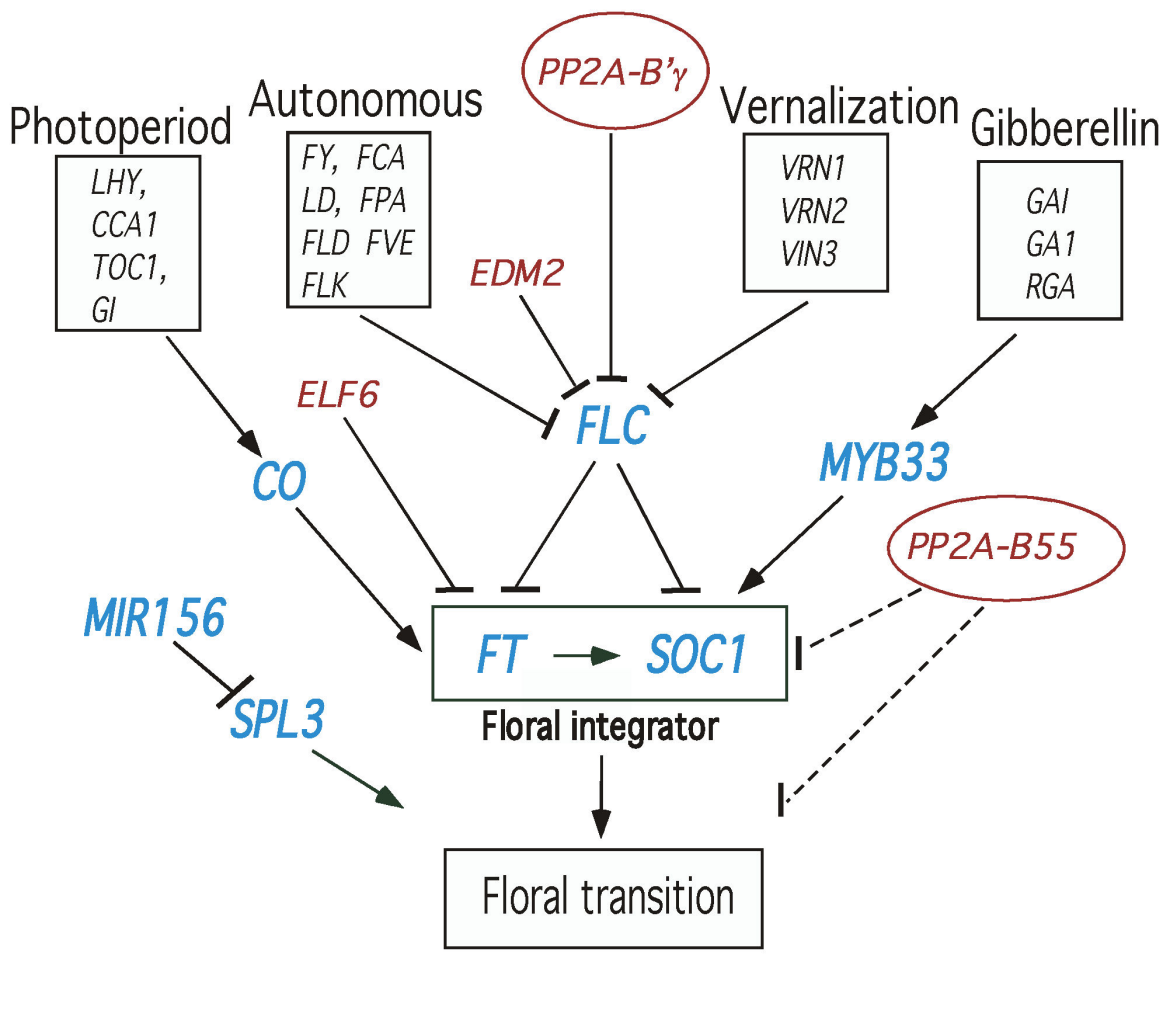
A schematic model for involvement of PP2A in flowering time pathways in *Arabidopsis* . Expression of key genes of each pathway, *CO*, *FLC*, and *MYB33* as well as *FT* and *SOC1* of the floral integrator were tested. *SPL3* and *miR156*, which can influence flowering by an endogenous pathway acting downstream of the floral integrator were also tested. *ELF6* and *EDM2* genes are known to delay and advance flowering, respectively. Mutants (knockout) lines of *elf6* and *edm2* were included as control lines. Genes (transcripts) tested in this work are shown in blue. Genes mutated in *Arabidopsis* lines tested in this work are shown in red. The work showed that *PP2A-B55* was a negative regulator of flowering time possibly acting downstream of/at the floral integrator, whereas *PP2A-B’γ* was a positive component in flower induction acting through modulation of *FLC* expression.

So far four major flowering time signalling pathways are acknowledged: the photoperiod, autonomous, vernalization, and gibberellin pathway (Figure 1) [13–15]. A more complete picture would include also pathways from ambient temperature, nitrate status, and age signals [16,17]. Genes named here refer to *Arabidopsis* , but orthologous are generally found in other plants studied [18–20]. The photoperiod pathway regulates flowering time in response to external signals, especially length of the photoperiod. This pathway involves for example the photoreceptors phytochrome and cryptochrome, clock genes, and further downstream the zinc-finger transcription factor *CO* (*CONSTANS*). *CO* promotes flowering through *FT* (*FLOWERING LOCUS T*) and SOC1 (SUPPRESSOR OF OVEREXPRESSION OF CONSTANS 1). *FT* together with *FD* (a bZIP transcription factor) and *SOC1* up-regulate various genes, including *AP1* (*APETALA1*), and change the vegetative meristem (VM) into an inflorescence meristem (IM) that in turn rise to a floral meristem (FM) [17]. Other pathways responding to environmental signals are the vernalization pathway and ambient temperature pathway. In the vernalization pathway, *FLC (FLOWERING LOCUS C)*, the main flowering repressor gene, is inactivated by chromatin modifications in response to enduring cold periods. The requirement for vernalization in the “lab strains” like Columbia is, however, abolished due to a mutation in another gene (*FRIGIDA*) [17]. *FLC* also has a central place in the autonomous pathway, but external signals are not important for induction of flowering in the pathways termed autonomous [13]. A positive effect of gibberellin on flowering is seen especially in short day plants and biennial plants under non-inductive conditions. *MYB33* is considered as an important flowering promoting component of the gibberellin pathway [21,22]. In the age pathway a specific subset of *SPL* (*SQUAMOSA PROMOTOR BINDING PROTEIN-LIKE*) genes is an important activator. The microRNA miR156 is an inhibitor of *SPLs*, and *MIR156* expression decreases with age [23]. The *miR156/SPL* pathway may act also downstream of *FT* on flowering, hence forming a pathway independent of the important *FT* (florigen) gene [24].

Phosphorylation/dephosphorylation of proteins is recognized as essential for mediating light signals through phytochromes and cryptochromes to the photoperiodic pathway. Components downstream of the photoreceptors, e.g. circadian clock components, are also known to depend on phosphorylation status for proper function [25–27]. Another component of the photoperiodic pathway, CO, binds regulatory 14-3-3 proteins, which generally interact with specific phosphorylated motifs indicating that phosphorylation status of CO can be important [28]. Concerning the autonomous/vernalization pathway it has been pointed out that FLC is a phosphorylated protein, and that phosphorylation inhibits the activity of FLC, hence promoting early flowering [29]. In the shoot, FT and phosphorylated FD which are downstream components of all four major flowering pathways, form a complex with 14-3-3 called florigen activating complex, and initiate transcription of genes important for inducing the flower producing meristem [28,30]. These examples from the literature show that phosphorylation is crucial in the transition from the vegetative to the floral state in plants. To further elucidate this important developmental transition and identify the signalling pathways where PP2As are involved, we used *Arabidopsis* mutated in PP2A regulatory subunits B55 and B’. We recorded flowering time under various conditions, and examined expression of central genes in genetically distinct flowering pathways and the floral integrator. The results showed that *PP2A-B55* is a negative regulator of flowering, whereas *PP2A-B’γ* is a positive regulator of flowering that contributes to the repression of the major flowering repressor gene *FLC* in *Arabidopsis* .

## Materials and Methods

### Plant material and growth

T-DNA insertion lines provided by SALK [31] and GABI-Kat [32] were obtained from NASC (The European Arabidopsis Stock Centre in Nottingham, UK). *Arabidopsis* mutant lines for *bα* (AT1g51690) were SALK_032080 (insertion in second exon) and SALK_095004 (insertion in sixth intron). Mutant lines for *bβ* (AT1g17720) were SALK_062514 (insertion in sixth intron) and GK_290G04 (insertion in fifth exon). Mutant line for *b*’ γ were SALK_039172 (insertion in 5’ UTR) and a *pp2ab’γ* line complemented by 35S-driven expression of the PP2A-Bγ gene (in SALK_039172 background) [12].

Mutant selection was done by PCR using primers for T-DNA insertion lines recommended by SALK institute website SIGnAL (http://signal.salk.edu/tdnaprimers.2.html). Homozygous mutants were verified by PCR using gene specific primers [11]. Known flowering time mutants used as controls were *edm2* (AT5g55390, SALK_014520C) and *elf6-3* (AT5g04240, SALK_074694C). Seeds were sown in a regular plant soil mixture, stratified at 4^o^C for 2 days, and then transferred to growth chambers with 8 h/16 h, 12 h/12 h, 16 h/8 h light/darkness or continuous light.

For testing of expression levels of flowering regulatory genes, seeds were stratified for 4 days at 4^o^C before placed in a 16 h light/8 h dark regimen. Shoots were harvested 10 days after germination, and generally 12 h into the photoperiod. In one experiment shoots were also harvested 8 h into the photoperiod, which confirmed the results.

### Phenotyping

Plants were observed daily. Number of rosette leaves and flowering time were recorded. Flowering time was measured using two different time points: first, appearance of the floral bud (DTF1) as indicator of transition from vegetative to inflorescence meristem, and second, appearance of first open flower (DTF2) as indicator of transition from inflorescence meristem to floral meristem [33,34]. Fresh weight of leaves was measured 21 days after germination to assure that observed phenotype is not simply due to altered growth rate in mutant plants. Characterizing of flowering phenotypes was repeated at least three times and in successive generations for each mutant line to assure that observations are repeatable and phenotypes are stable during generations.

### qRT-PCR

Quantitative reverse transcriptase real time PCR (qRT-PCR) was performed as previously described [35]. Total RNA was isolated using RNeasy® Plant Mini Kit (Qiagen, Chatswort, CA), and cDNA synthesised using the High Capacity cDNA Archive Kit (Applied Biosystems). MicroRNA was isolated using mirVana miRNA isolation Kit (Invitrogen) and cDNA was made using the TaqMan MicroRNA reverse transcription kit (Applied Biosystems). Real-time PCR reactions were assayed using an ABI 7300 Fast Real-Time PCR System. The reaction volume was 25 µL containing 12.5 µL TaqMan buffer (Applied Biosystems, includes ROX as a passive reference dye), 8.75 µL H_2_O, 2.5 µL cDNA and 1.25 µL primers. Primers were predesigned TaqMan® Gene Expressions assays obtained for the following *Arabidopsis thaliana* genes, (Applied Biosystems identification number is given in parenthesis): *FT* At1g65480 (At02224075), *FLC* At5g10140, (At02272498), *MYB33* At5g06100 (At02337117), *SOC1/AGL20* AT2g45660 (At02263356), *CO* At5g115840, (At02200179), SPL At2g33810 (At02204412), MIR156A At2g25095 (Assay ID000333). The Taq Man assay is based on a light signal from each transcript copy formed and allows comparing expression levels between the various genes. Standard cycling conditions (2 min at 50° C, 10 min at 95° C and 40 cycles altering between 15 s at 95° C and 1 min at 60° C) were used for product formation. Real-time PCR products were analyzed by Sequence Detection Software version 1.3. Comparative CT method for relative quantification has been used with ubiquitin At3g02540 (At02163241_g1), ACT8 At1g49240 (At02270958) and SnoR85 (Assay id 0017111) as endogenous controls. Relative quantity (RQ=2^-ΔΔCT^) of any gene in mutant lines was calculated relative to WT (calibrator). Expression levels are given as per cent of WT, which was set to 100%.

## Results

**Figure 2:**
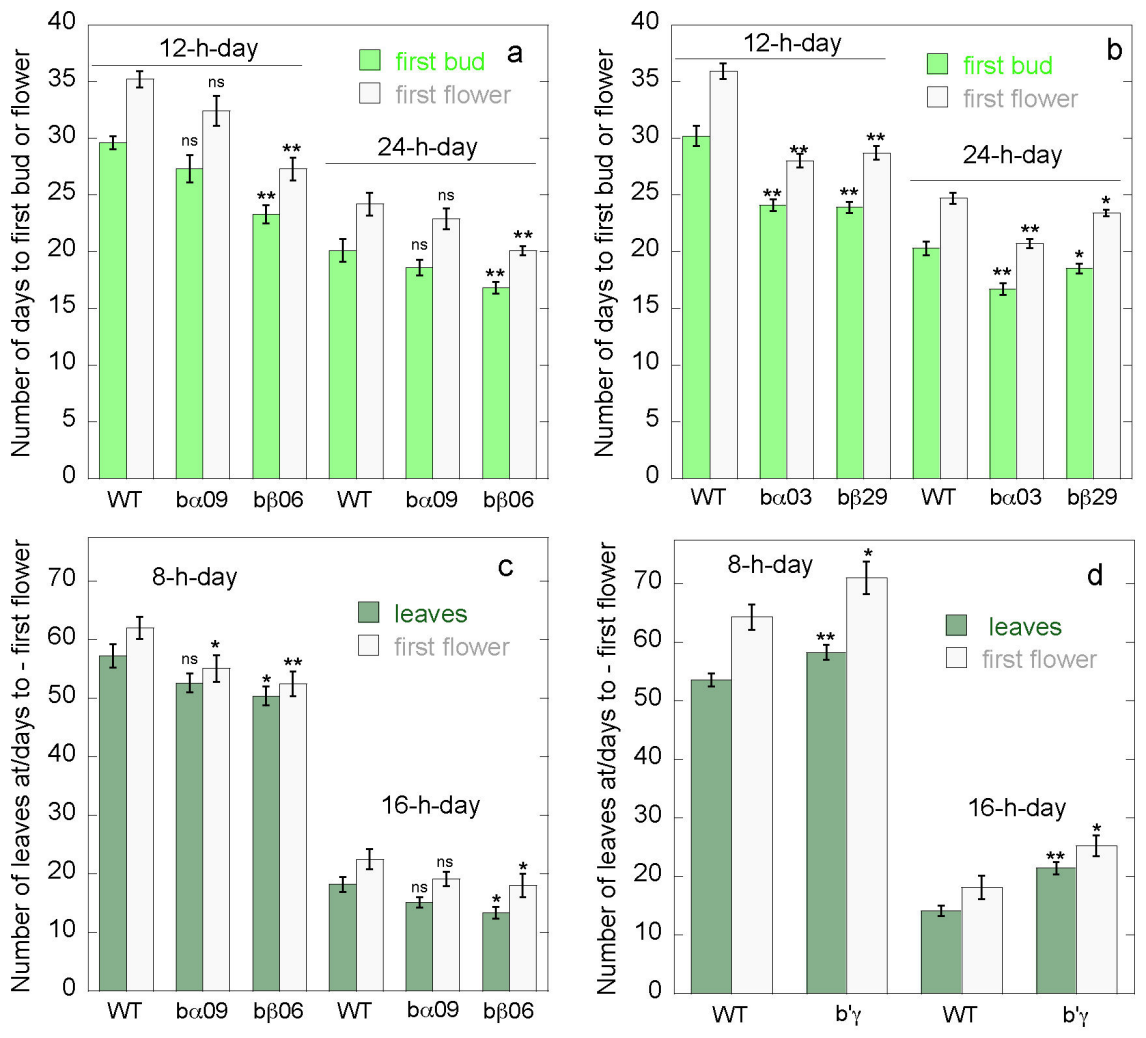
Flowering time for WT, *pp2a-bα*, *pp2a-bβ* and *pp2a-b’γ* lines grown in 8, 12, 16 or 24 h days. **a**) Days until first bud (green columns) and first flower (white columns) for WT, *pp2a-bα* (SALK_09504), *pp2a-bβ* (SALK_062614) in 12 h and 24 h days. **b**) Days until first bud (green columns) or first flower (white columns) for WT, *pp2a-bα* (SALK_032080), *pp2a-bβ* (GK_ 290G04) in 12 h and 24 h days. **c**) Numbers of leaves at first flower (dark green columns) and number of days to first flower (white columns) for WT, *pp2a-bα* (SALK_09504), *pp2a-bβ* (SALK_062614) in 8 h and 16 h days. **d**) Numbers of leaves at first flower (dark green columns) and number of days to first flower (white columns) for WT, and *pp2a-b’γ* (SALK_039172) in 8 h and 16 h days. The data show that the four *pp2a-b55* mutant lines tested were early flowering, and the *pp2a-b’γ* mutant line was late flowering under all conditions tested. For each genotype and treatment 30 plants were scored. SE is given. Columns marked with one or two stars are significantly different from WT at p < 0.05 and p < 0.01, respectively (student’s t-test).

**Figure 3:**
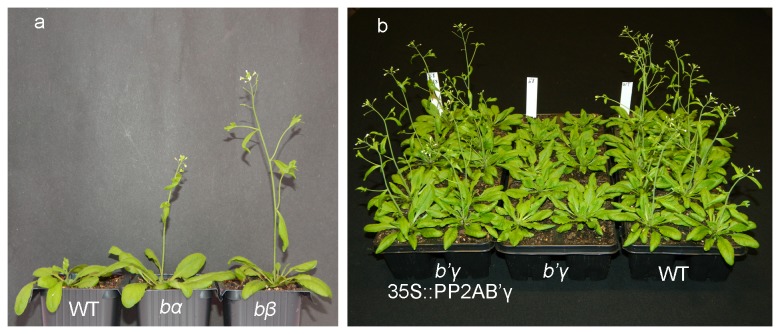
Visible phenotypes of representative *pp2a* mutants. **a**) Representative plants of WT and early flowering mutant lines *pp2a-bα* (SALK_09504) and *pp2a-bβ* (SALK_062614). **b**) Mutant line *pp2a-b’γ* complemented with 35S::PP2A-B’γ showing flowering time as WT, late flowering line *b’γ* (SALK_039172), and WT plants. Plants were grown in 16 h days.

Expression analysis had previously verified that transcripts of *PP2A-B55α* and *PP2A-B55β* genes were not detectable in the respective mutants [11] and transcript level of *PP2A-B’γ* was low in the *pp2a*-*b’γ* mutant plants [12]. The *PP2A-B’’γ* complementation line contains variable levels of mRNA (2.2-fold ± 1.0-fold compared with wild-type levels) [12]. Plants were first grown in 12 h days or continuous light to test number of days to flowering (Figure 2a, b). Averaged across all four *pp2a-b55* mutant lines in 12 h days, the first bud appeared 5.2 days, and the first flower 6.6 days before WT. In continuous light the first bud appeared 2.5 days and the first flower 3.4 days before WT. *pp2a-b55* mutant lines were also observed in 8 and 16 h days, where the first flower appeared 8.2 and 4 days before WT, respectively (Figure 2c). The mutant line *pp2a-b’γ* was observed in 8 h and 16 h days, and showed that the first flower appeared 7.1 (in 8 h days) and 4.7 (in 16 h days) days later than in WT (Figure 2d). Early or late floral transition is reflected also by the formation of lower or higher number of rosette leaves prior to bolting. Compared with WT, *pp2a-b55* plants formed nearly four and six rosette leaves less before bolting, under long and short day conditions, respectively (Figure 2c). In contrast, plants mutated in *pp2a-b’γ* formed nearly seven and five more rosette leaves before bolting compared with WT, under long and short day conditions (Figure 2d). In conclusion all *pp2a-b55* mutant lines tested were early flowering, and the *pp2a-b’γ* mutant line was late flowering under all conditions. The *pp2a*-*b55* mutants showed growth very similar to WT. In 12 h days or continuous light the number of leaves was the same for mutant lines and WT 21 days after sowing. In 12 h days, fresh weight of leaves was also nearly identical for mutants and WT. The *pp2a-bα* line showed lower fresh weight in continuous light compared with WT and *pp2a-bβ* (Table 1). The results imply that the early flowering of the *pp2a-b55* mutants is not caused simply by increased growth. On the contrary, these mutants showed same or slower growth compared with WT, hence the criteria for qualifying as a real flowering time mutant are strengthened [13]. Figure 3 illustrates the early flowering phenotype of *pp2a-b55* and late flowering phenotype of *pp2a-b*’*γ*.

**Table 1:** Growth of WT, *PP2A-b55α*, and *PP2A -b55β*.

	**12 h photoperiod**		**24 h photoperiod**	
**Genotype**	**Number of leaves**	**mean leaf weight**	**Number of leaves**	**mean leaf weight**
WT	7.7 ± 0.6	0.13 ± 0.02	7.9 ± 0.7	0.18 ± 0.04
*pp2a-b55α* SALK_09504	7.2 ± 0.6	0.12 ± 0.02	7.2 ± 0.5	0.13 ± 0.02
*pp2a-b55β* SALK_062614	7.2 ± 0.6	0.13 ± 0.02	7.1 ± 0.6	0.18 ± 0.02

Number of leaves per plant 16 days after germination, and mean leaf fresh weight 21 days after germination. For each genotype and treatment 45 plants were scored. SE is given.

**Figure 4:**
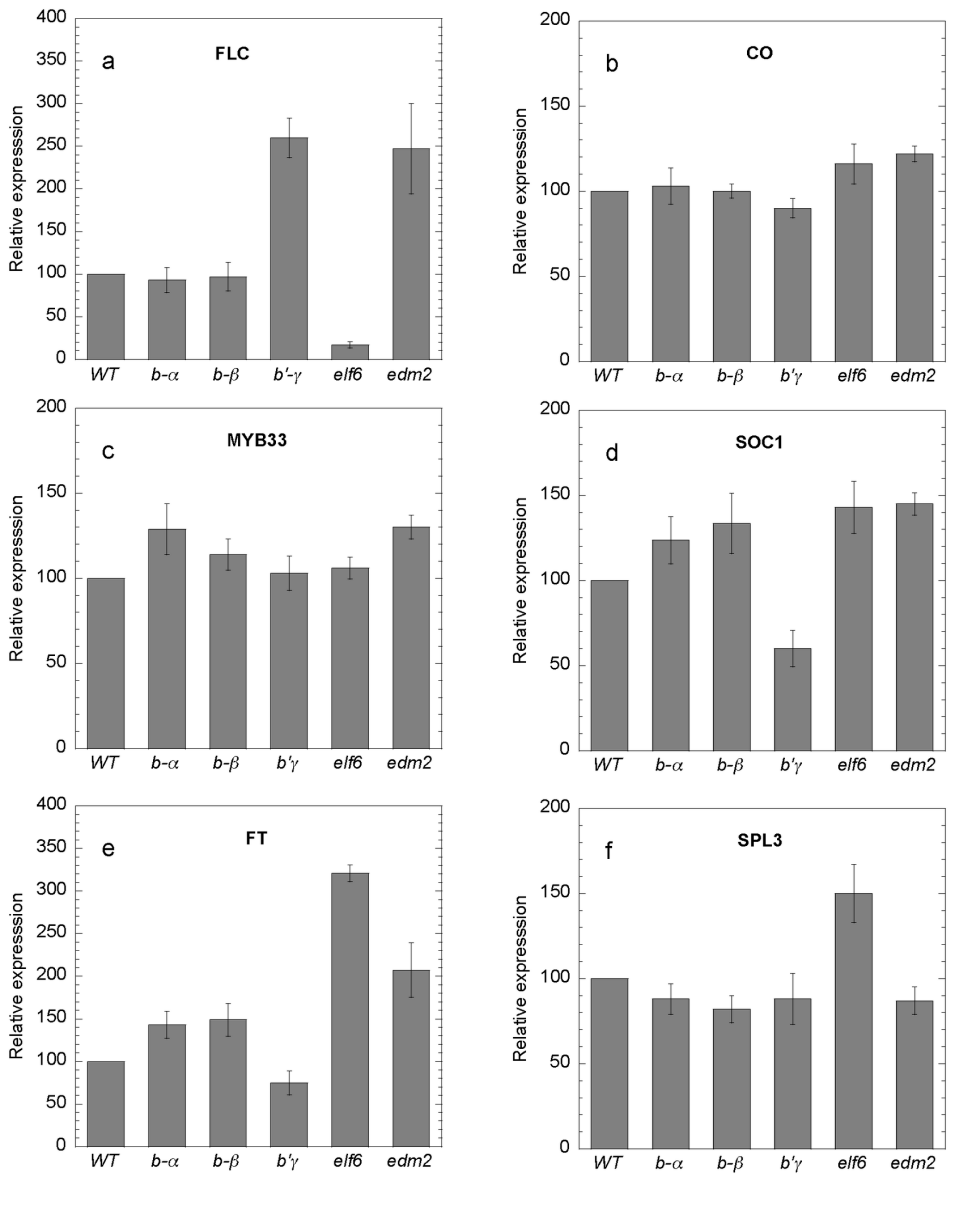
Expression levels of genes important in different flowering time controlling pathways and the floral integrator. Shoots were harvested ten days after germination, 12 h into the 16 h photoperiod. Gene expression was tested in WT and the mutants *pp2a-bα SALK_09504*, *pp2a-bβ SALK_062614*, *pp2a-b’γ*, e*lf6* (early flowering control) and *edm2* (*late flowering control*). Genes tested were: **a**) *FLC*, **b**) *CO*, **c**) *MYB33*, **d**) *SOC1*, **e**) *FT* and **f**) *SPL3*. Expression of established flowering pathway genes are modulated in *pp2a-b’γ* consistent with this mutant being late flowering, whereas *pp2a-b55* mutants show only minor changes in transcript levels and may act on flowering time through an unknown pathway. Data presented are means of three (except for *SPL3*, which had two) independent experiments of samples each containing 50 plants and assayed in triplicate. Vertical bars indicate the standard error of the mean.

**Figure 5:**
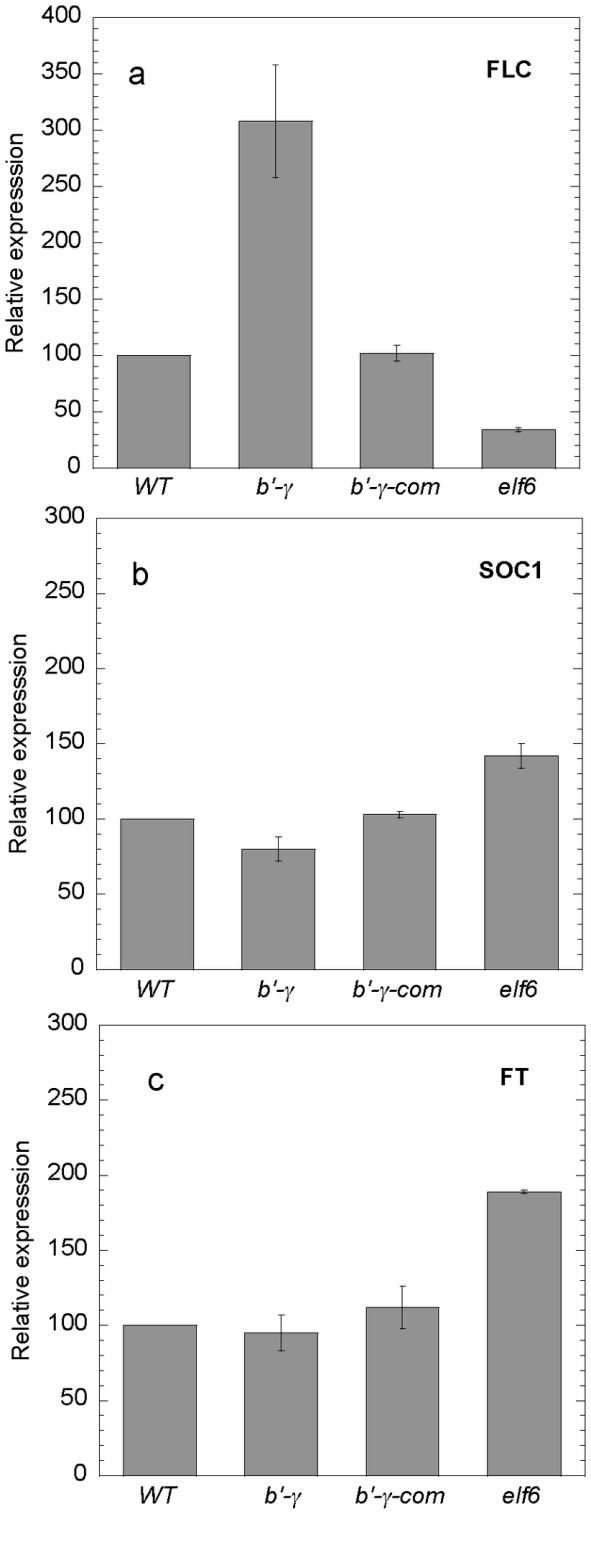
Expression levels of flowering control genes after complementation of the *pp2a-b’γ* mutant. Shoots were harvested ten days after germination, 12 h into the 16 h photoperiod. Gene expression was tested in WT, and the mutants, *pp2a-b’γ, pp2a-b’γ-complemented*, and e*lf6* (control). Genes tested were: **a**) *FLC*, **b**) *SOC1*, and **c**) *FT*. The *pp2a-b’γ* mutant showed normal WT expression levels when complemented with the *35S-PP2A-B’γ* gene construct. Data presented are means of three independent experiments for *FLC* expression and two for *SOC1* and *FT*. Each sample contained 50 plants and was assayed in triplicate. Vertical bars indicate the standard error of the mean.

Known flowering mutants were included in the experiments to assure the relevance of the expression analysis for detecting flowering time mutants in the various signalling pathways. *EDM2* has a promoting effect on flowering, and acts upstream of the floral repressor *FLC*, in other words *EDM2* represses *FLC* [36]. *ELF6* is an inhibitor of flowering, and delays flowering through the photoperiod pathway [37] by repressing *FT* [38], as well as through an autonomous pathway by effects on brassinosteroid signalling components [39]. Expression analysis of these genes showed that the late flowering mutant control, *edm2*, had high level of *FLC* expression whereas the early flowering mutant control, *elf6*, showed low level of *FLC* and high level of *FT* expression (Figure 4a, e). Low level of *FLC* transcripts in the *elf6* mutant was not previously reported, but a close relative to *ELF6* (*REF6*) was found to inhibit flowering through the autonomous pathway by acting on *FLC* [37]. Interestingly, in our analysis using the highly specific TaqMan assays, it was clear that *FLC* was expressed at a low level also in the *elf6* mutant (Figure 2a). For *pp2a-b55α/β* mutants, expression levels of *FLC* and *CO* were the same as in WT, but there was a tendency to slightly higher expression levels of the floral integrator genes *FT* and *SOC1*. A particular microRNA, miR156, is known to promote the juvenile phase in *Arabidopsis* and maize and inhibit flowering [13]. Recently, a new autonomous pathway was pointed out where *MIR156* and *SPL3* play important roles [24]. Since these genes can act downstream of *FT* and *FD* we tested *SPL3* and *MIR156* expression in the *pp2a* mutants. However, expression of *SPL3* was not significantly altered in *pp2a* mutants (Figure 4f). Expression of *MIR156* was also tested (data not presented), but average deviation from WT was only 30% (down-regulated). In conclusion, the SPL3/miR156 pathway could hardly explain early flowering time in *pp2a-b55* mutants. Consistent with late flowering phenomena, gene expression analysis in the *pp2a-b’γ* mutant line showed high levels of *FLC* and low levels of *FT* and *SOC1* expression compared with WT (Figure 4). Complementation of *pp2a-b’γ* with the *PP2A-B’γ* gene under control of the 35S promoter restored WT expression of *FLC*, *FT*, and *SOC1* (Figure 5), and the complemented line flowered as early as WT (Figure 3b) e.g. average flowering time was 20.3 ± 0.7 and 20.0 ± 0.7 days for WT and complemented line, respectively. The results are consistent with *PP2A-B’γ* promoting flowering in *Arabidopsis* through repression of the *FLC* gene.

## Discussion

### Multilevel control of *Arabidopsis* flowering time by protein phosphoregulation

A high degree of phosphorylation of proteins in the photoperiodic pathway, as well as the florigen activating complex, generally has been associated with acceleration of flowering [25,27,28], e.g. high kinase/low phosphatase activity would promote flowering. Knockout of a protein phosphatase interacting with the photoperiodic pathway or florigen activating complex, could therefore lead to earlier flowering than for WT. Although their specific protein targets are still unknown, a kinase promoting flowering, CKII, as well as a kinase that delays flowering, SNF1-like AKIN10, have been identified [40,41]. The phosphorylation status of proteins is a result of opposing kinases and phosphatases, and late flowering of the *pp2a-b’γ* knockdown mutant would be consistent with PP2A-B’γ acting antagonistically to a kinase that delays flowering. Furthermore, PP2A plays a role in brassinosteroid signalling, and the B'γ subunit (among other B’subunits) interacts with a key component, BZR, of the signalling pathway [7]. Impairment of brassinosteroid signalling is known to result in delayed flowering [39]. Delayed flowering in *pp2a-b’γ* knockdown may therefore also potentially (partly) act through changes in a brassinosteroid influenced pathway. By testing expression of selected genes of the main flowering pathways we intended to clarify which pathways PP2A subunits would target.

### Photoperiod, Gibberellin and Vernalization pathways

The *pp2a-b55α* and *pp2a-b55β* mutants showed early flowering compared with wild type in continuous light, 16 h, 12 h and 8 h photoperiods (Figure 2). Mutations in the genes of the photoperiod regulatory pathway make *Arabidopsis* unable to sense the duration of inductive long photoperiods, resulting in altered flowering time in long days, but little effect in short days [13,37]. *pp2a-b55* mutants flower earlier than wild type whatever the photoperiod, but since they are responsive to photoperiod and are able to discriminate between short and long days and still flower earlier in long days than they do in short days, mutations in the *PP2A-B55α or PP2A-B55β* genes are not likely to interfere specifically with the photoperiod pathway. This was also supported by the fact that expression of *CO* was not altered in *pp2a*-*b55* mutants in comparison with WT. Flowering time of the *pp2a-b’γ* mutant was also perturbed (delayed) in both short and long days but the mutant is also able to discriminate between short and long days which indicates that *B*’ γ is also not a candidate for being involved in the photoperiodic pathway. The gibberellin pathway is mainly of importance in short days [21,22], which implies that the *pp2a-b55α, pp2a-b55β* and *pp2a-b'γ* mutations did not interfere specifically with this pathway since the phenotypes were pronounced regardless of length of the photoperiod. Furthermore, we did not find any large changes in expression of *MYB33*, a flowering promoting gene in the gibberellin pathway [21]. The vernalization pathway is generally inactivated through the *FRIGIDA* mutation in the *Arabidopsis* *Columbiae* line, therefore the vernalization pathway is not a candidate for being involved in our experiments [13].

### Autonomous pathway

In the autonomous pathway the flowering inhibitor *FLC* plays an important role. In the late flowering control mutant *edm2* expression of *FLC* was clearly enhanced in agreement with the autonomous pathway being involved (Figure 4a) [36]. This confirmed that our marker genes and expression analysis would reveal flowering time mutants of this pathway. Since expression analysis of *FLC* in the *pp2a-b55α* and *pp2a*-*b55β* mutants did not show any deviations from expression in WT, the classical autonomous pathway is unlikely to account for early flowering of the *pp2a-b55* mutants. However, increased phosphorylation status of the FLC protein itself still represents one possible mechanism of PP2A effects and cannot be completely ruled out, although enhanced *FT* transcript level should follow inactivation of FLC. *FT* transcripts, however, showed only a very moderate increase in *pp2a-b55*. In the *pp2a-b’γ* mutant, transcript level of the *FLC* gene was three-fold higher than in WT, which implies that *PP2A-B’γ* plays an important role in the repression of *FLC*, and knockdown of *PP2A-B’γ* results in a late flowering phenotype. The results are consistent with *PP2A-B’γ* being a component of the autonomous pathway upstream of *FLC*. *FLC* is epigenetically regulated [13] and a PP2A scaffolding subunit was recently found to interact with histone deacetylase HDA14 [42]. In further work it will be interesting to explore if PP2A-B’γ is part of a histone modifying complex.

In conclusion, based on observations with different photoperiods and the fact that the vernalization pathway is not important in the Columbia line, three of the main flowering pathways, photoperiod, vernalization, and gibberellin pathway, are not likely to be targets for *PP2A-B55* or *PP2A-B’γ*. *PP2A-B55* may target components at the downstream level of floral integrators through an unknown pathway, but targeting of different pathways should not be excluded. *PP2A-B’γ* functions in the autonomous pathways by repressing the main flowering inhibitor *FLC*. The results show that PP2A acts both as a positive and negative regulator of flowering, depending on the regulatory B subunit involved.
